# Group, Subgroup and Person‐Specific Longitudinal Associations Between Physical Activity and Affect in Individuals With and Without Depressive and Anxiety Disorders

**DOI:** 10.1002/mpr.70085

**Published:** 2026-06-19

**Authors:** Noa van Zwieten, George Aalbers, Femke Lamers, Harriëtte Riese, Manon H. J. Hillegers, Brenda W. J. H. Penninx

**Affiliations:** ^1^ Department of Psychiatry Amsterdam UMC Vrije Universiteit Amsterdam Amsterdam the Netherlands; ^2^ Amsterdam Public Health Mental Health Program Amsterdam the Netherlands; ^3^ Department of Psychiatry Clinical Cognitive Neuro‐Psychiatry (CCNP) University Medical Center Groningen University of Groningen Groningen the Netherlands; ^4^ Department of Child and Adolescent Psychiatry/Psychology Erasmus University Medical Center Rotterdam the Netherlands

**Keywords:** affect, ambulatory assessment, anxiety, depression, physical activity

## Abstract

**Objectives:**

Associations between physical activity and affect (activity‐affect dynamics) vary among individuals for which reasons remain unclear. We examined whether such heterogeneity is explained by psychiatric status or sociodemographic, clinical, and ambulatory assessment characteristics.

**Methods:**

Two‐week ambulatory assessment data of 300 participants with current (*n* = 79), subthreshold (*n* = 67), and no (*n* = 154) depressive and/or anxiety disorders or symptoms were obtained from the Netherlands Study of Depression and Anxiety. Positive and negative affect (PA/NA) were assessed with ecological momentary assessment (5xdaily) and physical activity using actigraphy. Group iterative multiple model estimation was used to model associations shared across the sample, psychiatric subgroups, and those specific to individuals.

**Results:**

No activity‐affect associations were shared across the sample (i.e., present in > 75% of all individuals) or psychiatric subgroups (i.e., present in > 51% of individuals within subgroups). Nevertheless, 45% of participants had at least one activity‐affect association, with considerable heterogeneity in their nature. The most frequent association was a positive contemporaneous association between physical activity and PA (present in 25% of the sample).

**Conclusions:**

These findings suggest large heterogeneity in activity‐affect dynamics among individuals and underscore the importance of considering the unique dynamics of the individual.

## 1 Introduction

Depressive and anxiety disorders are prevalent mental disorders associated with substantial disability (Vos et al. 2016) and limited treatment outcomes (Gaynes et al. 2009). Consistent evidence, also from our group, has linked lower daily levels of physical activity to depressive and anxiety disorders (Burton et al. 2013; Difrancesco et al. 2019; Hiles et al. 2017). One potential mechanism underlying this association is the regulatory role of physical activity in maintaining mood homeostasis (Chan et al. 2019), with mood being a sustained emotional state, closely linked to momentary affect (Trzepacz and Baker 1993). Given this regulatory role, physical activity is used as sole or adjunctive treatment to prevent or treat depressive (Hearing et al. 2016) and anxiety symptoms (Ensari et al. 2015). Meta‐analyses report heterogeneous and small to modest effect sizes of physical activity interventions for depressive and anxiety disorders, but comparable to other treatments such as medication or psychotherapy (Hu et al. 2020; Noetel et al. 2024; Stubbs et al. 2017). The effectiveness of physical activity interventions is often evaluated by comparing groups, assuming homogeneity which might mask individual variations (Kanning et al. 2013), potentially limiting the success of such interventions. Collectively, these findings warrant a deeper understanding on the complex dynamics between physical activity and affect (i.e., activity‐affect dynamics).

There has been a growing recognition of using ambulatory assessments, such as ecological momentary assessment (EMA) and actigraphy, in studying activity‐affect dynamics in daily life (Doherty et al. 2017; Trull and Ebner‐Priemer 2009). EMA involves the repeated assessment of daily affective states within individuals. Actigraphy objectively measures physical activity with a wearable, usually worn around the wrist or at the hip. Previous research using ambulatory assessment has shown that physical activity is both directly and longitudinally associated with improved positive affect (PA) in individuals with depressive and anxiety disorders, although substantial heterogeneity has been observed (Difrancesco et al. 2022; Stavrakakis et al. 2015; Wichers et al. 2012). This heterogeneity may suggest that not all individuals benefit equally from physical activity. For instance, some individuals engage in physical activity to “feel better” (i.e., regulating affect by increasing PA and lowering negative affect (NA)), bolstering future physical activity behavior, whereas for others being physically active may lead to increased NA (Ekkekakis and Brand 2019). That is why we cannot understate the importance of exploring person‐specific activity‐affect dynamics, using the so‐called idiographic research approach (Molenaar 2004).

Idiographic research not only endeavors to explore how dynamics differ across individuals, but also to identify subgroups of individuals with similar dynamics. Such subgroup identification might be useful for clinical practice, as it might allow us to provide physical activity interventions to individuals most likely to benefit from them (Ruissen 2022), an important objective of precision medicine (Collins and Varmus 2015). Additionally, from a theoretical perspective, subgroup identification evaluates if the mood‐regulatory function of physical activity is compromised in individuals with depressive and anxiety disorders. A statistical approach well‐suited for exploring associations at individual, subgroup, and group levels is Group Iterative Multiple Model Estimation (GIMME; Gates and Molenaar 2012). GIMME estimates individual and group‐level models showing how variables are related across time without assuming within‐group homogeneity (Gates and Molenaar 2012). GIMME is also capable of identifying subgroups theory‐driven, based on predefined subgroups (CS‐GIMME; Henry et al. 2019) and completely data‐driven, based on similar patterns of associations (S‐GIMME; Gates et al. 2017). Hence, GIMME bridges idiographic and nomothetic approaches by enabling generalization at the group and subgroup level while accommodating heterogeneity at the individual level (Gates and Molenaar 2012).

At present, no studies have investigated to what extent person‐specific activity‐affect associations cluster into meaningful subgroups nor if such subgroups are related to psychiatric status. To this end, we used confirmatory CS‐GIMME and data‐driven S‐GIMME to analyze a unique clinical dataset of 300 individuals with current, subthreshold, and no depressive and/or anxiety disorders or symptoms that combines EMA of PA and NA (5 times daily for 14 days) with research‐grade actigraphy. In an earlier analysis of these data (Difrancesco et al. 2022), we found physical activity to be associated with subsequent improvement in PA although there was substantial heterogeneity, requiring further research. Building on this, our pre‐registered study's (https://osf.io/yu4cf, Zwieten et al. 2023) first objective was to explore if activity‐affect associations were shared by the full sample or predefined psychiatric status‐based subgroups, or unique for individuals. Based on our previous findings (Difrancesco et al. 2022), we hypothesized that individuals with current depressive and/or anxiety disorders would show stronger positive associations between physical activity and PA than individuals with subthreshold or no depressive and/or anxiety disorders and symptoms (Hypothesis 1). Our second objective was to identify putative data‐driven subgroups of individuals with similar patterns of associations and examine if such subgroups relate to sociodemographic, clinical and, ambulatory assessment characteristics. We hypothesized that GIMME would identify data‐driven subgroups (Hypothesis 2), but given the exploratory nature of this aim, we did not have hypotheses about their relation to sociodemographic, clinical and ambulatory assessment characteristics.

## 2 Method

### 2.1 Participants

This study is a secondary data analysis on the data from the NESDA EMA & Actigraphy (NESDA‐EMAA) sub‐study, previously described in other publications (Difrancesco et al. 2019, 2022; Schoevers et al. 2021). Participants of the NESDA‐EMAA sub‐study were selected from the 9‐year follow‐up of the NESDA study. The NESDA study is an ongoing longitudinal multi‐site cohort study intended to examine the long‐term course of depressive and anxiety disorders. Further details about the NESDA study design and inclusion procedures are provided in other sources (Penninx et al. 2008, 2021). For the current study, participants were included at baseline in 2004–2007 if they were Dutch‐fluent adults between 18 and 65 years (*n* = 2981) and completed the fifth assessment wave at 9‐year follow‐up in 2014–2017 (*n* = 1776). Additionally, 367 siblings of NESDA participants were added to the fifth wave (*n* = 2143). In total 384 participants enrolled in the EMAA sub‐study as previously described and shown in the flowcharts for the EMA (Schoevers et al. 2021) and actigraphy part (Difrancesco et al. 2019). The NESDA study, including the EMAA sub‐study, was approved by the VUmc ethical committee (reference number 2003/183). All participants gave informed consent for the regular assessment and the EMAA sub‐study.

Participants filled out EMA questionnaires five times a day with a three‐hour fixed design on their phones and wore an actigraphy device (GENEactiv, Activinsights Ltd.) on their non‐dominant wrist for 14 consecutive days. Participants were instructed to complete questionnaires as soon as possible after receiving the text message, preferably within 15 min, but at least within 60 min, and received a reminder after 30 min. Data were gathered with our secured server system (RoQua; Sytema and Van Der Krieke 2013). A smartphone was provided to participants with no smartphone or no suitable smartphone (e.g., no internet bundle; *n* = 107, 27.9%). After data cleaning (see Difrancesco et al. 2019; Schoevers et al. 2021 for more details) 359 participants had valid EMA and actigraphy data. Building on this sample of 359 participants we performed three additional data cleaning steps in line with GIMME recommendations (Lane et al. 2019). First, we excluded 50 participants who did not complete at least 60 (86%) of the maximal 70 possible EMA assessments in order to recover accurate models, as recommended by Lane et al. (2019). Second, two participants were excluded from analyses as they had no variance on the NA subscale and GIMME does not converge when variance on at least one of the variables in the model is lacking (Lane and Gates 2017). Third, after conducting a preliminary GIMME analysis, we removed seven individuals with unusually high beta coefficients (|β| > 1) and standard errors (> 1), as we considered their models as unreliable. This criterion was applied separately for each GIMME extension, resulting in the exclusion of seven participants and a final analytic sample of *n* = 300. The excluded participants (*n* = 59) did not differ from the final analytic sample (*n* = 300) in sociodemographic characteristics (age, sex, education, employment status) or psychiatric status (all *p*'s > 0.05), suggesting that the excluded sample did not differ systematically from the included sample.

Simulation studies have shown that GIMME with subgrouping yields reliable results with as few as 25 individuals in the total group (Gates et al. 2017) and is robust with as few as 60 observations per individual (Lane et al. 2019). With a minimum of 60 timepoints per individual and 300 individuals we believe we had sufficient power to perform the analyses.

### 2.2 Measures

#### 2.2.1 Psychiatric Status

Psychiatric status was defined using the Composite International Diagnostic Interview (CIDI version 2.1; Wittchen 1994), the Inventory of Depressive Symptomatology‐Self Report (IDS‐SR_30_; Rush et al. 1996), and the Beck Anxiety Index (BAI; Beck et al. 1988), all assessed at the 9‐year follow‐up. The CIDI assesses DSM‐IV diagnoses of depressive and anxiety disorders. The IDS‐SR_30_ and BAI assesses severity of depressive and anxiety symptoms.

Three groups were defined for confirmatory CS‐GIMME (H1): (1) individuals with current depressive and/or anxiety disorders, defined as a CIDI diagnosis within the past 6 months (*n* = 79), (2) individuals with subthreshold depression and/or anxiety disorders (*n* = 67), defined as not having a current diagnosis but having a high IDS‐SR_30_ score of ≥ 14 (cut‐off for mild depression) or a high BAI score of ≥ 11 (cut‐off for mild anxiety symptoms), and (3) individuals with no disorders or symptoms (*n* = 154), defined as not having a current diagnosis and a IDS‐SR_30_ score ≤ 13 and a BAI score ≤ 10.

#### 2.2.2 Sociodemographic and Clinical Characteristics

To describe and compare subgroups, we included several sociodemographic and clinical characteristics assessed at the 9‐year follow‐up questionnaire assessment and interview session.

##### 2.2.2.1 Sociodemographic Characteristics

We included age, sex (male, female), employment status (employed, not employed) and education level in years.

##### 2.2.2.2 Clinical Characteristics

Depressive and anxiety symptom severity were defined based on the IDS‐SR_30_ (Rush et al. 1996) and the BAI (Beck et al. 1988), respectively. Psychiatric history was categorized into remitted (those with a past but not current psychiatric diagnosis) and healthy controls (those without a lifetime diagnosis). The number of psychiatric disorders calculated as a count of current depressive and anxiety diagnoses at 9‐year follow‐up. Analogous to Difrancesco et al. 2019; Schoevers et al. 2021, the duration of depressive and anxiety disorders was calculated as a count of the number of waves at which the patients reported depressive and/or anxiety disorders during the in‐between follow‐up periods (ranging from one to five waves). Antidepressant and benzodiazepine use (based on drug container inspection) was included if participants reported using it more than 50% of days in a month. Medications were coded according the World Health Organization Anatomical Therapeutic Chemical (ATC) classification. We included selective serotonin reuptake inhibitors (SSRIs, ATC code N06AB), tricyclic antidepressants (TCA, ATC code N06AA) and other antidepressants (ATC codes N06AF, N06AG, N06AX); benzodiazepines included ATC codes N03AE, N05BA, N05CD, and N05CF. The number of chronic somatic diseases was included and was based on a 21‐item face‐to‐face interview designed for the NESDA study. The total number of diseases for which persons received treatment was calculated. Finally, body mass index was included (BMI; kg/m^2^).

#### 2.2.3 Ambulatory Assessment Characteristics

##### 2.2.3.1 Daily Hassles and Uplifts

To describe and compare subgroups, we included the total number of daily hassles and uplifts which were evaluated using the EMA item (assessed 5 times a day): *Did you have daily (un)pleasant experiences since you filled out the previous assessment?* The answer options were: *yes something pleasant, yes something unpleasant, yes both something pleasant and unpleasant, and none.* The number of daily uplifts were counted as reported pleasant experiences, and the number of daily hassles as reported unpleasant experiences.

##### 2.2.3.2 Physical Activity

The accelerometer was set to sample at 30 Hz. In line with Difrancesco et al. 2019, we preprocessed raw actigraphy data using the R GGIR package version 3.0‐2 (Hees et al. 2013; Migueles et al. 2019). We defined physical activity using the acceleration metric Euclidian Norm Minus One g with negative values rounded to zero (1g = 9.81 m/s2; ENMO), as this metric correlates well with daily energy expenditure (Hees et al. 2013).

##### 2.2.3.3 Momentary Positive and Negative Affect

Momentary PA and NA states were assessed with EMA 5 times a day, including 13 momentary affect items from the Uncovering the Positive Potential of Emotional Reactivity Study (Bennik 2015). Momentary affect items were rated on a 7‐point Likert scale ranging from “1 = not at all” to “7 = very much”. A PA subscale was calculated as the average of PA items (*at this moment I feel satisfied, relaxed, cheerful, energetic, enthusiastic, calm*) and a NA subscale as the average of NA items (*at this moment I feel upset, irritated, listless/apathic, down, nervous, bored, anxious*).

### 2.3 Statistical Analyses

#### 2.3.1 Data Preprocessing

We aggregated actigraphy data based on the EMA survey timestamps, averaging ENMO between the corresponding timestamp of completing the EMA survey and 3 hours prior for all EMA surveys. If the participant missed an EMA assessment (5.2%) resulting in a missing completion timestamp, the scheduled timestamp for sending out the EMA questionnaire was used. Next, following previous work (Bringmann et al. 2016; Kullar et al. 2024), we excluded overnight lags from the analysis by inserting a row of missing values between the final EMA survey of the previous day and the first of the present day. Hence, auto‐ and cross‐regressive associations are based on within‐day lags only. As GIMME assumes weak stationarity of the data (Beltz and Gates 2017), we removed linear trends per person and per variable over the 14 day‐period.

#### 2.3.2 Statistical Testing

**FIGURE 1:**
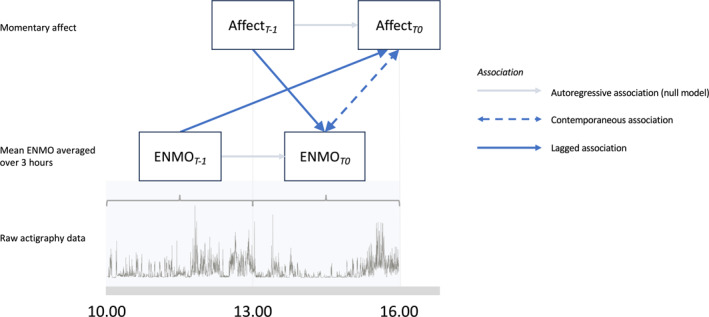
Temporal Design of GIMME models, illustrating lagged and contemporaneous associations between momentary affect assessed at EMA timepoints and physical activity measured via actigraphy (ENMO). Raw actigraphy data (5‐s epochs) are shown for illustration. ENMO was calculated by averaging the raw acceleration signal over the 3‐h time window prior to each EMA timepoint, resulting in a single mean ENMO value per timepoint (ENMO_*T–1*_, ENMO_*T0*_).

The analyses deviate in some aspects from the pre‐registered plan on https://osf.io/yu4cf (Zwieten et al. 2023), described in the Supporting Information S1. To model associations between ENMO, PA, and NA we used the R package *gimme* (version 0.7‐15; Lane et al. 2023). GIMME estimates person‐specific network models, mapping both lagged and contemporaneous associations among variables based on unified Structural Equation Modeling (uSEM; Gates et al. 2017; Gates and Molenaar 2012). Given the aggregation of actigraphy data based on EMA timepoints, lagged and contemporaneous associations have specific temporal interpretations (see Figure 1 for details). Lagged effects represent either the association between ENMO averaged over the 3 h prior to the *previous* EMA timepoint (e.g., 10:00–13:00) and affect at the current EMA timepoint (e.g., 16:00), corresponding to a 3–6 h window (ENMO_t–1_ → PA_t0_), or between affect assessed at the previous EMA timepoint (e.g., 13:00) and current ENMO that was averaged over the 3 hours prior to the current EMA timepoint (e.g., 13:00–16:00), corresponding to 0–3 h window (e.g., PA_t–1_ → ENMO_t0_). Contemporaneous effects represent the association between affect assessed at a given EMA timepoint (e.g., 16:00) and ENMO averaged over the 3 h prior to that same timepoint (e.g., 13:00–16:00), corresponding to a 0–3 h window (e.g., PA_t0_—ENMO_t0_).

Two specific extensions of GIMME were used. First, for testing H1 we used confirmatory CS‐GIMME (Henry et al. 2019) with three psychiatric status subgroups as described before to examine whether activity‐dynamics are shared by individuals within these subgroups. Second, for testing H2 we used data‐driven S‐GIMME (Gates et al. 2017) to examine whether subgroups of individuals with similar dynamic processes could be identified. Both analyses were run with the same, mainly default settings, described in the Supporting Information S1.

GIMME operates iteratively, beginning with a null model for each individual that includes only autoregressive effects, followed by the estimation of group, subgroup, and individual‐level associations in that order. The group‐level cut‐off was set at 75% and the subgroup‐level cut‐off was set at 51%, both recommended in the literature (Beltz and Gates 2017; Gates and Molenaar 2012; Lane et al. 2019). These cut‐offs mean that an association is included in the group or subgroup‐level network if it significantly improves model fit for the majority of the individuals in the group (i.e., > 75%) or subgroup (i.e., > 51%). In the last step, GIMME estimates individual‐level associations which are added to a person‐specific model if they significantly (*p* < 0.05) improve model fit for the participant until excellent model fit is achieved according to at least two of four fit indices: comparative fit index (CFI) ≥ 0.95; non‐normed fit index (NNFI) ≥ 0.95; root mean squared error of approximation (RMSEA) ≤ 0.05; standardized root mean residual (SRMR) ≤ 0.05. The final person‐specific models thus show group‐ and subgroup‐level associations and individual‐level associations that are significant for the individual.

To evaluate the robustness of both CS‐GIMME and data‐driven S‐GIMME subgroupings we examined the modularity value *Q*, reflecting the strength of a network into subgroups (Gates et al. 2017). Values for networks with robust and strong subgroup structures typically range from 0.3 to 0.7 (Newman and Girvan 2004). To further evaluate the robustness of subgroupings with positive *Q* values, we performed validity checks following Gates et al. (2019), described in the Supporting Information S1. If validity checks confirmed the robustness of subgroups, we compared them on sociodemographic, clinical, and ambulatory assessment characteristics using chi‐square tests and ANOVAs. In the absence of robust subgroup solutions, we explored the person‐specific activity‐affect associations based on their frequency. To probe our data further, we explored if individuals with the most frequent person‐specific activity‐affect association differed from those without this association using chi‐square tests and independent *t*‐tests. We considered group differences statistically significant if their *p*‐values were below 0.05 after False Discovery Rate correction for multiple testing (Benjamini and Hochberg 1995). The analysis code is available on https://osf.io/bjdy6/. Data were analyzed using R, version 4.2.3 (R Core team, 2023).

## 3 Results

### 3.1 Participants

The mean age of the participants was 50.3 years (SD = 12.1) and 190 participants (63.3%) were female. According to the structured psychiatric assessment, 79 individuals had a current depressive and/or anxiety disorder, 67 individuals had a subthreshold depressive and/or anxiety disorder, and 154 individuals had no disorder or symptoms (Beck et al. 1988; Rush et al. 1996; Wittchen 1994). The participants completed on average 66.3 valid EMA assessments (SD = 2.9) and had 69.9 valid actigraphy entries (SD = 0.8). The descriptive statistics of the sociodemographic, clinical, and ambulatory assessment characteristics per subgroup are given in Table 1.

**TABLE 1:** Sociodemographic, clinical and, ambulatory characteristics for psychiatric status subgroups (*n* = 300).

Subgroups according to psychiatric assessment (CIDI, IDS, BAI)	Current depressive and/or anxiety disorders	Subthreshold depressive and/or anxiety disorders	No disorders or symptoms	*p‐*value
*n* (%)	79 (26.3)	67 (22.3)	154 (51.3)	
Sociodemographic characteristics				
Age (years), mean (SD)	50.4 (10.8)	51.4 (12.5)	49.7 (12.5)	0.613^a^
Female, *n* (%)	48 (60.8)	49 (73.1)	93 (60.4)	0.206^b^
Education level (years), mean (SD)	12.3 (3.3)	12.6 (3.0)	13.2 (2.9)	0.024^a^
Employment status, *n* (%)	39 (49.4)	29 (43.3)	104 (67.5)	0.002^b^
Clinical characteristics				
Current anxiety disorders only, *n* (%)	30 (38.0)	—	—	
Current depressive disorders only, *n* (%)	21 (26.6)	—	—	
Comorbid depressive & anxiety disorders, *n* (%)	28 (35.4)	—	—	
Psychiatric history, *n* (%)	—	66 (98.5)	84 (54.5)	
Duration of disorders (number of waves), median (IQR)	4 (1)	2 (2.5)	1 (2)	< 0.001^a^
Severity of depressive symptoms (IDS), mean (SD)	25.3 (13.1)	20.7 (7.1)	6.1 (3.5)	< 0.001^a^
Severity of anxiety symptoms (BAI), mean (SD)	13.0 (9.4)	9.2 (6.5)	2.6 (2.6)	< 0.001^a^
BMI (kg/m^2^), mean (SD)	27.1 (5.2)	27.1 (5.6)	26.4 (5.2)	0.351^a^
Number of chronic diseases, mean (SD)	0.9 (1.0)	1.1 (1.1)	0.6 (0.7)	0.012^a^
Psychotropic medication use				
Antidepressant users, *n* (%)	30 (38.0)	20 (29.9)	11 (7.1)	< 0.001^b^
Benzodiazepine users, *n* (%)	4 (5.1)	5 (7.5)	3 (1.9)	0.179^b^
Ambulatory assessment characteristics				
Sum of daily uplifts, median (IQR)	16.0 (19.5)	19.0 (20.0)	19.0 (22.8)	0.216^a^
Sum of daily hassles, median (IQR)	8.0 (7.0)	7.0 (10.0)	5.0 (6.8)	0.011^a^
ENMO (mg × 1000), mean (SD)	33.6 (24.8)	35.0 (23.7)	37.5 (23.5)	< 0.001^a^
Positive affect, mean (SD)	4.1 (1.1)	4.5 (1.0)	5.3 (0.9)	< 0.001^a^
Negative affect, mean (SD)	2.2 (1.0)	1.7 (0.8)	1.3 (0.5)	< 0.001^a^

*Note:* FDR‐corrected significant differences are indicated by *p* < 0.05. ENMO values have been multiplied by 1000 for ease of interpretation.

Abbreviations: IQR = interquartile range, SD = Standard deviation.

^a^ Analysis of Variance (ANOVA).

^b^ *χ*2 test of independence.

**FIGURE 2:**
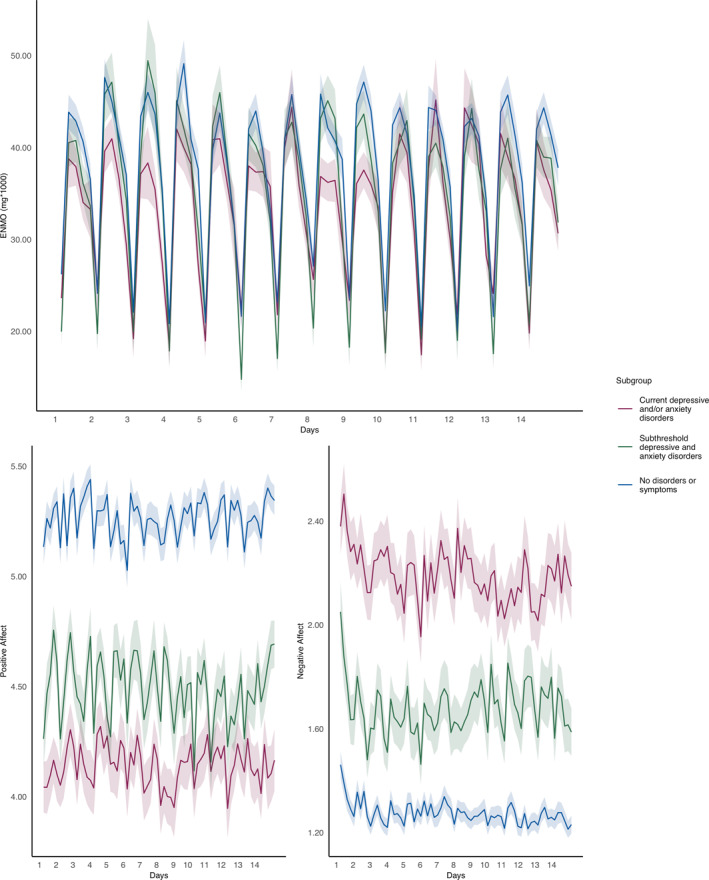
Aggregated mean levels (lines) and standard errors (shaded areas) of (a) ENMO, (b) positive affect, and (c) negative affect over the course of 14 days per psychiatric status subgroup. Note that the *y*‐axis for Figures 2b and 2c differ although they were assessed on the same scale—see methods section for details. ENMO values have been multiplied by 1000 for ease of interpretation.

Individuals with current depressive and/or anxiety disorders had significantly higher scores on depressive and anxiety symptom severity (*p* < 0.001) and had a higher frequency of antidepressants use (*p* < 0.001), but not benzodiazepine use (*p* = 0.179), compared to the other groups. Compared to individuals with current or subthreshold disorders, those without disorders or symptoms had significantly higher educational attainment (*p* = 0.024), higher rates of employment (*p* = 0.002), and fewer chronic diseases (*p* = 0.012). Additionally, the three subgroups significantly differed in their mean levels of ENMO, PA, and NA with individuals with current depressive and anxiety disorders having the lowest mean levels of PA and ENMO, and highest level of mean NA (*p*'s < 0.001). The mean ENMO, PA, and NA per group over time are depicted in Figure 2.

### 3.2 GIMME

#### 3.2.1 CS‐GIMME for Confirmatory Subgroups

CS‐GIMME was performed with three confirmatory subgroups based on psychiatric status. A total of 296 (out of 300) person‐specific models fit the data well according to the mean fit indices (RMSEA = 0.02, NNFI = 0.64, CFI = 0.99, SRMR = 0.06).^1^ The models of four individuals did not meet the criteria for excellent fit (RMSEA = 0.09, NNFI = 0.40, CFI = 0.90, SRMR = 0.10). These four individuals were excluded for further analyses. All detected associations are given in Table 2.

**TABLE 2:** The total number of individuals and mean beta coefficients per detected association between ENMO, PA, NA for confirmatory CS‐GIMME (*N* = 296).

			Frequency			Beta coefficient			
Association			Total *n* ^a^	*n* positive beta^b^	*n* negative beta^b^	Mean	SD	Min	Max
Group									
NA_*t*_	—	PA_*t*_	296	10	286	−0.513	0.254	−0.936	0.537
Person‐specific									
ENMO_*t*_	—	PA_*t*_	77	75	2	0.319	0.161	−0.512	0.619
NA_*t–1*_ ^c^	→	PA_*t*_	21	12	9	0.042	0.372	−0.640	0.487
PA_*t–1*_	→	ENMO_*t*_	20	14	6	0.183	0.405	−0.440	1.048
ENMO_*t*_	—	NA_*t*_	20	13	7	0.179	0.440	−0.409	1.179
PA_*t–1*_ ^c^	→	NA_*t*_	19	4	15	−0.380	0.449	−0.932	0.487
ENMO_*t–1*_ ^c^	→	NA_*t*_	17	7	10	−0.017	0.379	−0.412	0.895
NA_*t–1*_ ^c^	→	ENMO_*t*_	11	6	5	0.030	0.426	−0.482	0.522
ENMO_*t–1*_ ^c^	→	PAt_t0_	10	6	4	0.064	0.318	−0.376	0.436

*Note:* Contemporaneous associations identified by GIMME are presented without directional interpretation and aggregated accordingly. The person‐specific association between PA and NA was omitted due to a significant group‐level association. Rows are ordered by frequency of detected associations.

Abbreviations: NA = Negative Affect, PA = Positive Affect, SD = standard deviation of the beta coefficient.

^a^ Total *n* indicates the total number of individuals for whom each association is included in the model.

^b^ *n* positive beta and *n* negative beta indicate the number of individuals with positive and negative associations, respectively.

^c^ Underscript t–1 and arrows denote a lagged association.

##### 3.2.1.1 Group‐Level Results

One group‐level contemporaneous association between NA and PA was found, which had a mean beta coefficient of −0.51 (SD = 0.25) and was significant for 88.2% of the individuals with converged models. This indicates that for most individuals increased NA was associated with decreased PA at the same timepoint. We did not find other group‐level associations.

##### 3.2.1.2 Subgroup‐Level Results

No subgroup‐level associations were found, indicating there were no associations between ENMO, PA, and, NA that were shared by the majority (i.e., > 51%) of each predefined subgroup. The modularity value *Q* was negative (*Q* = −0.004), indicating that the subgroup solution was not robust. There was no support for psychiatric status‐based subgrouping in estimating associations among ENMO, PA, and NA.

##### 3.2.1.3 Individual‐Level Results

In the absence of activity‐affect associations at the group and subgroup level, GIMME estimated many unique individual‐level associations. As given in Table 2, the associations vary widely in strength (i.e., beta coefficients), sign (i.e., positive or negative), timing (i.e., contemporaneous and lagged) and direction. The models of 134 out of 296 individuals (45.3%) contained at least one significant activity‐affect (PA or NA) association (*p <* 0.05), with the most common association being the positive contemporaneous association between ENMO and PA estimated in 75 individuals (25.3%). This association had a mean beta coefficient of 0.34 (SD = 0.10). Of these 134 individuals with at least one activity‐affect association, 32 individuals had a current depressive and/or anxiety disorder, 30 individuals had a subthreshold depressive and/or anxiety disorder, and 72 individuals had no disorders or symptoms. Regarding the timing of all detected individual‐level activity‐affect associations, GIMME found more individuals with contemporaneous (32.1%) than lagged associations (17.2%). Moreover, we identified more individuals with individual‐level associations between physical activity and PA (33.8%) than NA (15.2%). To illustrate this heterogeneity a selection of person‐specific models is given in Supporting Information S1: Figure S1.

**FIGURE 3:**
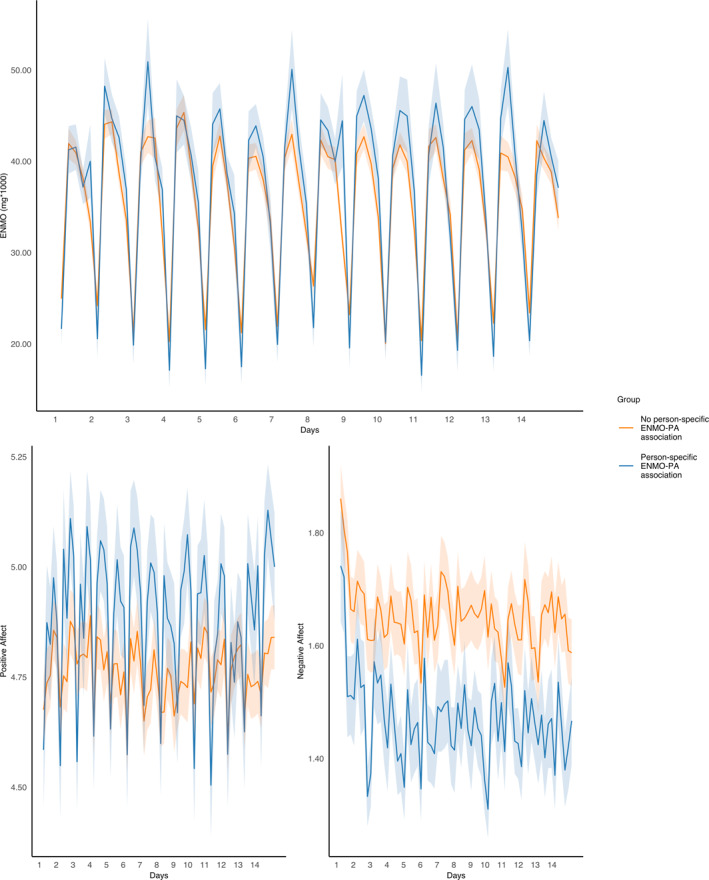
Aggregated mean levels (lines) and standard errors (shaded areas) of (a) ENMO, (b) positive affect, and, (c) negative affect over the course of 14 days for individuals with (*n* = 75) and without (*n* = 221) a positive contemporaneous association between ENMO and positive affect. Note that the *y*‐axis for Figure 3b and 3c differ although they were assessed on the same scale—see methods section for details. ENMO values have been multiplied by 1000 for ease of interpretation.

The positive contemporaneous association between ENMO and PA was the most commonly estimated individual‐level association. We therefore explored this association further by comparing individuals with this association (ENMO‐PA group; *n =* 75) to those without (no ENMO‐PA group; *n =* 221), to see if we could identify sociodemographic, clinical, or ambulatory assessment characteristics linked to this association. Descriptive statistics of both groups are given in Table 3. We found no differences on sociodemographic and clinical characteristics between the ENMO‐PA and no ENMO‐PA group. However, the groups significantly differed on PA, NA, and ENMO (*p*'s < 0.001), with the ENMO‐PA group having higher mean levels of PA and ENMO and lower NA. Additionally, the ENMO‐PA reported a higher number of daily uplifts than the no ENMO‐PA group (*p* = 0.039). These group differences are also depicted in Figure 3.

**TABLE 3:** Sociodemographic, clinical and, ambulatory characteristics for No ENMO‐PA and ENMO‐PA group (*n* = 296).

	No ENMO‐PA group	ENMO‐PA group	*p*‐value
*n* (%)	221 (74.6)	75 (25.3)	
Sociodemographic characteristics			
Age, mean (SD)	51.0 (12.1)	48.4 (11.8)	0.392^a^
Female, *n* (%)	136 (61.5)	52 (69.3)	0.637^b^
Education level (years), mean (SD)	12.8 (3.0)	12.9 (3.2)	0.970^a^
Employment status, *n* (%)	129 (58.4)	41 (54.7)	0.907^b^
Clinical characteristics			
Psychiatric status	—	—	0.510^b^
Current depressive and anxiety disorders, *n* (%)	65 (29.4)	14 (18.7)	—
Subthreshold depressive and anxiety disorders, *n* (%)	46 (20.8)	20 (26.7)	—
No disorders or symptoms, *n* (%)	110 (49.8.)	41 (54.7)	—
Psychiatric history, *n* (%)	106 (48.0)	41 (54.7)	0.703^b^
Duration of disorders (number of waves), median (IQR)	2 (3)	2 (4)	0.907^a^
Severity of depressive symptoms (IDS), mean (SD)	14.5 (11.9)	14.2 (11.3)	0.938^a^
Severity of anxiety symptoms (BAI), mean (SD)	7.0 (7.8)	6.2 (6.8)	0.703^a^
BMI, mean (SD)	26.6 (5.2)	27.0 (5.7)	0.907^a^
Number of chronic diseases, mean (SD)	0.8 (1.0)	0.8 (0.9)	0.938^a^
Psychotropic medication use			
Antidepressant users, *n* (%)	49 (22.8)	11 (14.7)	0.562^b^
Benzodiazepine users, *n* (%)	8 (3.6)	4 (5.3)	0.907^b^
Ambulatory assessment characteristics			
Sum of daily uplifts, median (IQR)	17.0 (21.0)	22.0 (24.0)	0.039^a^
Sum of daily hassles, median (IQR)	7.0 (8.0)	5.0 (7.5)	0.732^a^
ENMO (mg × 1000), mean (SD)	35.5 (22.9)	37.1 (26.8)	< 0.001^a^
Positive affect, mean (SD)	4.8 (1.1)	4.9 (1.0)	< 0.001^a^
Negative affect, mean (SD)	1.7 (0.9)	1.5 (0.6)	< 0.001^a^

*Note:* FDR‐corrected significant differences are indicated by *p* < 0.05. ENMO values have been multiplied by 1000 for ease of interpretation.

Abbreviations: IQR = interquartile range, SD = Standard deviation.

^a^ Independent *t*‐test.

^b^ *χ*2 test of independence.

#### 3.2.2 S‐GIMME for Data‐Driven Subgroups

S‐GIMME was performed to identify putative data‐driven subgroups that share associations between ENMO, PA, and NA. Similar to confirmatory CS‐GIMME, we did identify many individual‐level activity‐affect associations, but did not find these at the group or subgroup‐level. The modularity value of the subgroup solution was low (*Q* = 0.079) and additional tests to evaluate the robustness of the data‐driven subgroups did not point toward a robust subgroup solution (see Supporting Information S1). We preregistered to compare both confirmatory and data‐driven subgroups on various characteristics but deviate from this approach and only make these comparisons for the confirmatory subgroups, determined using gold‐standard clinical measures, as modularity values for both subgroupings indicate limited robustness. The results of the S‐GIMME associations at group, subgroup, and individual level are described in the Supporting Information S1.

## 4 Discussion

In the current study, we explored whether we could identify activity‐affect dynamics in individuals with and without depressive and/or anxiety disorders. More specifically, we used both a confirmatory and data‐driven subgrouping approach to examine whether psychiatric status subgroups could explain activity‐affect dynamics and whether different subgroups could be formed based on similar activity‐affect dynamics, respectively. Contrary to our hypotheses, our results yielded no activity‐affect dynamics that were shared by the majority of the sample, its subgroups based on psychiatric status (i.e., confirmatory approach), and similar dynamics (i.e., data‐driven approach). Nonetheless, we identified a positive contemporaneous association between physical activity and PA at the individual level in a minority of the sample (25%). Notably, individuals with this association did not share specific sociodemographic or clinical characteristics, but they had higher PA and ENMO and lower NA levels. Overall, this lack of group‐ and subgroup‐level associations highlights a considerable degree of heterogeneity within our rather large sample, suggesting that activity‐affect dynamics may vary widely across individuals.

Although psychiatric subgroups differed in mean affect and physical activity levels, consistent with previous findings in this sample (Minaeva et al. 2020), these between‐person differences did not translate into systematic differences in activity‐affect dynamics. The lack of consistent group or subgroup‐level activity‐affect associations and the presence of substantial variability suggest that these associations may be highly person‐specific rather than generalizable across individuals. These null findings may reflect fundamental differences between within‐person and between‐person analytical approaches (Kanning et al. 2013; Vuuregge et al. 2025), whereby aggregate‐level associations can mask substantial heterogeneity in temporal dynamics. Difrancesco et al. (2022) illustrate this in a previous study on the same dataset. Using generalized estimating equation (GEE) models, a population‐averaged approach that estimates group‐level associations without explicitly modeling individual‐level effects, they found that physical activity was associated with higher PA 3 hours later on between‐subject level. However, exploratory within‐subject analyses showed high variability regarding the presence and sign of person‐specific associations (Difrancesco et al. 2022).

Interestingly, in contrast to the Difrancesco et al. (2022) analyses in the same sample, we did not find a similar association between physical activity and affect on the group‐level at all. This discrepancy is likely explained by fundamental differences in analytical approach. In contrast to GEE models, GIMME employs a person‐specific approach that does not assume homogeneity (Henry et al. 2019). It identifies the best‐fitting model for each individual and only considers an association as shared when it reaches statistical significance for a predefined threshold of the sample (> 75% for group‐level, > 51% for subgroup‐level in this study). Nevertheless, in 45% of the individuals at least one association between physical activity and affect was identified, but these associations varied considerably in timing, direction, and sign. Such large heterogeneity has been demonstrated in previous research using ambulatory assessments and idiographic approaches (Rosmalen et al. 2012; Stavrakakis et al. 2015; Vuuregge et al. 2025). By employing an integrative approach using GIMME, we facilitated a more nuanced exploration of person‐specific activity‐affect dynamics and observed additional heterogeneity in the direction and timing of these associations, extending the findings of Difrancesco et al. (2022). Such integrative approaches that accommodate heterogeneity are crucial for identifying individuals who are most likely to benefit from physical activity based on their unique activity‐affect dynamics.

In an effort of identifying those who are more likely to benefit from physical activity, we propose three key insights based on our findings at the individual level to guide future research. First, individuals with a positive contemporaneous person‐specific association between physical activity and PA could be characterized with higher mean PA and ENMO and lower mean NA levels, but not with psychiatric status, sociodemographic, or clinical characteristics. These findings suggest that a positive association between physical activity and PA may signal subtle differences at the momentary level, but this does not extend to disparities in psychiatric status or stable characteristics. To further investigate this finding, future research may focus on identifying affective patterns (e.g., variability and instability) that potentially characterize individuals with a positive association between physical activity and PA. If such patterns can be detected, they could be leveraged in practice by using ambulatory assessment tools to monitor these patterns, ultimately to identify individuals and specific moments when physical activity is most likely to result in positive affective outcomes. Building on this, just‐in‐time adaptive interventions promoting physical activity could be designed to support individuals at key moments (Hardeman et al. 2019).

Second, when considering all individual‐level activity‐affect associations, we identified more contemporaneous (i.e., within same time window; 32%) than lagged associations (i.e., over time; 17%). Although the quantification and characterization of temporal aspects of activity‐affect dynamics were beyond the scope of our study, these aspects could provide clinical targets for interventions. That is, for individuals whose affect‐enhancing effects of physical activity are lost quickly, an intervention boosting physical activity may be less preferable over others (Wichers et al. 2012). Future research could advance this line of research by employing a design where changes in physical activity trigger the assessment of affect with EMA, enabling to better capture real‐time activity‐affect dynamics (Kanning et al. 2013). This may convey more fine‐grained insights on the temporal specificity of activity‐affect dynamics and hold promise for gaining a deeper understanding of how long the affect‐enhancing effects of physical activity last. This knowledge could potentially be leveraged in practice by targeting physical activity interventions to those who experience longer‐lasting benefits from physical activity.

Third, we found fewer associations between physical activity and NA (15%) than PA (34%), corresponding to studies and reviews providing little and inconsistent evidence on the association between physical activity and NA in healthy (Kanning et al. 2013; Liao et al. 2015; Timm et al. 2024; Vuuregge et al. 2025) and depressed individuals (Mata et al. 2012; Stavrakakis et al. 2015; Wichers et al. 2012). This could be due to floor effects regarding NA (i.e., low NA levels) present in our sample, especially in individuals with no disorders and symptoms (von Klipstein et al. 2023), and could render it difficult to estimate activity‐affect associations. Nonetheless, the differentiation of affective states in relation to physical activity warrants more research as this may guide tailored interventions targeting specific affective outcomes. For example, individuals who tend to engage in physical activity to “feel better” (Ekkekakis and Brand 2019), particularly those with high levels of NA, may require other intervention strategies compared to those who engage in physical activity with high levels of PA (Ekkekakis et al. 2005; Stevens et al. 2020). Similarly, providing physical activity interventions to those who repeatedly experience higher NA after being physically active may be not effective at all and might require behavioral interventions other than physical activity (e.g., social activities that do not involve sports).

The study had major strengths as well as limitations. Strengths include the relatively large sample and the innovative GIMME analysis allowing for person‐specific insights on both objective and self‐report data on physical activity and affect. Limitations include the aggregation of contemporaneous associations as we considered the direction of these associations as not interpretable. GIMME for multiple solutions (GIMME‐MS), an extension within GIMME, allows for a better examination of the directionality of these associations (Beltz and Molenaar 2016), but is not yet available in combination with both subgrouping extensions. Second, we used a continuous measure of physical activity (ENMO) instead of categorizing physical activity into different levels (e.g., light, moderate‐to‐vigorous) and types (e.g., aerobic, house‐chore related activity). While ENMO provides a good measure of overall physical activity, it does not capture the distinct effects of different activity intensities and types on affect or the specific thresholds at which effects occur (Li et al. 2022). Therefore, future research should incorporate both activity levels and types to gain a more nuanced understanding of the context of activity‐affect dynamics. Third, in absence of group‐ and subgroup‐level associations, we interpreted the individual‐level associations, but the stability of such associations within GIMME may be less reliable than associations at the group and subgroup level (Siepe and Heck 2023). With a longer study period, thus increasing the number of timepoints per person, these individual‐level associations may be more reliable.

## 5 Conclusions

To conclude, the current study sheds light on the complex and multifaceted activity‐affect dynamics in individuals with current depressive and/or anxiety disorders, subthreshold depression and/or anxiety disorders, and those without disorders or symptoms. We used 2‐week ambulatory assessment data on physical activity and affect and found that activity‐affect dynamics could not be captured by subgroups, highlighting the large heterogeneity present in these dynamics. Moving forward, future research is warranted to further elucidate the mechanisms of activity‐affect dynamics. By leveraging the therapeutic potential of activity‐affect dynamics, physical activity interventions can be personalized and tailored to the individual.

## Author Contributions

**Noa van Zwieten:** conceptualization, formal analysis, writing – original draft. **George Aalbers:** writing – review and editing, supervision. **Femke Lamers:** funding acquisition, project administration, writing – review and editing, supervision. **Harriëtte Riese:** writing – review and editing, project administration. **Manon H. J. Hillegers:** funding acquisition, writing – review and editing, supervision. **Brenda W. J. H. Penninx:** funding acquisition, writing – review and editing, supervision.

## Funding

This work is funded by Stress in Action. The research project ‘Stress in Action’: www.stress‐in‐action.nl is financially supported by the Dutch Research Council and the Dutch Ministry of Education, Culture and Science (NWO gravitation grant number 024.005.010).

## Ethics Statement

The NESDA study and the EMAA sub‐study were approved by the VUmc ethical committee (reference number 2003/183).

## Consent

All participants gave informed consent for the regular assessment and the EMAA sub‐study.

## Conflicts of Interest

The authors declare no conflicts of interest.

## Permission to Reproduce Material From Other Sources

The authors have nothing to report.

## Supporting information


Supporting Information S1

## Data Availability

According to European law (GDPR) data containing potentially identifying or sensitive patient information are restricted; our data involving clinical participants are not freely available in a public repository. However, data are available upon request via the NESDA Data Access Committee (nesda@amsterdamumc.nl).

## Endnotes

^1^
The NNFI fit index of one individual was extremely low (−128), causing the mean NNFI fit index of 0.64 to be lower than what is qualified as excellent fit (NNFI > 0.95). However, the other fit indices for this individual were excellent, hence we did not exclude this individual.
