# Supplementary material for: Group, Subgroup and Person‐Specific Longitudinal Associations Between Physical Activity and Affect in Individuals With and Without Depressive and Anxiety Disorders
Source: Int J Methods Psychiatr Res. 2026 Jun 19;35(2):e70085. doi: 10.1002/mpr.70085 (PMC13282464; doi:10.1002/mpr.70085)
Supplement: Supplementary file 1 — Supporting Information S1 [file MPR-35-e70085-s001.docx]

**Supporting Information**

**Group, subgroup and person-specific longitudinal associations between physical activity and affect in individuals with and without depressive and anxiety disorders: An ambulatory assessment study**

Noa van Zwieten^1,2*^, dr. George Aalbers^1,2^, dr.ir. Femke Lamers^1,2^, dr. Harriëtte Riese^3^, prof.dr. Manon H.J. Hillegers^4^, prof.dr. Brenda W.J.H.Penninx^1,2^

1. Department of Psychiatry, Amsterdam UMC, Vrije Universiteit Amsterdam, Amsterdam, the Netherlands.
2. Amsterdam Public Health, Mental Health Program, Amsterdam, The Netherlands
3. University of Groningen, University Medical Center Groningen, Groningen, Department of Psychiatry, Clinical Cognitive Neuro-Psychiatry (CCNP), the Netherlands.
4. Department of Child and Adolescent Psychiatry/Psychology, Erasmus University Medical Center, Rotterdam, The Netherlands.

^*^Correspondence should be addressed to Noa van Zwieten; n.vanzwieten@amsterdamumc.nl

## Supplementary Methods

### Deviations from pre-registered analytic plan

The aims and analytic plan were pre-registered on <https://osf.io/yu4cf> (Zwieten et al., 2023). The analyses deviated from the pre-registered plan in a couple of aspects. First, regarding sample size we specified in the pre-registered plan that we would analyze the sample of *n* =359. However, we did not consider the GIMME recommendations specifying that more than 60 observations per person are preferable (Lane et al., 2019) for robust estimates of variable associations. Hence, we excluded 50 participants who did not complete at least 60 EMA observations, as recommended by (Lane et al., 2019) and as described in the Methods section. Additionally, we did not specify that we would exclude individuals with unusually high beta coefficients (|β| > 1) and standard errors (>1) after conducting a preliminary analysis of both CS-GIMME and S-GIMME (for details see the Method section). Second, as described in the Method section, we initially planned to compare both confirmatory and data-driven subgroups on sociodemographic, clinical, and ambulatory assessment characteristics. However, because modularity values for both CS- and S-GIMME indicated limited robustness of subgroup solutions, we deviate from our preregistered approach and only make these comparisons for the confirmatory subgroups, which we determined using gold-standard clinical measures. Third, we had not preregistered that in case no subgroup-specific associations were identified either by confirmatory CS-GIMME or completely data-driven S-GIMME, we would more closely interpret person-specific associations. Specifically, we described and compared individuals with the most predominant person-specific association between physical activity and affect to those without this association using chi-square tests for categorical characteristics and independent t-tests for continuous characteristics.

**Additional Information on GIMME**

### GIMME default settings

Both confirmatory CS-GIMME and completely data-driven S-GIMME were mainly run with default settings, autoregressive paths at the group level were freed (i.e., for all individuals in the sample; Lane et al., 2019), subgroups were based on the Walktrap community detection algorithm (Pons & Latapy, 2005), and the inference criteria for pruning of paths were set with a Bonferroni correction of *α*= .05/*N,* where *N* is the number of individuals (Lane & Gates, 2017). Data was standardized within GIMME. Missing data was handled within GIMME using full information maximum likelihood (FIML).

### Evaluation of subgroup stability

A positive *Q* value suggests a stronger and robust subgroup structure (i.e., a stronger indication of the presence of subgroups), whereas a negative *Q* value may indicate that the subgroup solution is not robust (i.e., less reliable). To further evaluate the robustness of the subgroups with positive *Q* values, we performed validity checks using the R package *perturbR* (Gates et al., 2019). Following Gates et al., (2019) subgroups were considered as robust if 1) the modularity value was greater than or equal to the 95^th^ percentile in a distribution of random perturbed matrices, and 2) 20% or more of the edges could be perturbed before 20% of the participants were allocated into different subgroups compared to the original subgroup solution (Karrer, Levina, & Newman, 2008). The latter criterium was evaluated with the Variation of Information (VI) metric and the Adjusted Rand Index (ARI), both reflecting the degree of differences between the original solution and the distribution by randomly swapping 20% of cluster assignments.

## Supplementary Results

### Sample diagrams of individual-level models from confirmatory CS-GIMME

Four sample network model plots are presented in Figure S1. These network models visualize the heterogeneity between physical activity and affect present in our sample. As there was a contemporaneous negative association between PA and NA at the group level, this association has been added to all the person-specific models. For participant 4 (Figure S1a), there was a positive and contemporaneous association between PA and ENMO. Note that although the direction as indicated in the figure is from PA to ENMO, we do not interpret the directions for the contemporaneous associations as we consider them not interpretable. This association indicates that this individual experiences higher PA when being physically active (or the opposite). This association is not present in the network models of the other participants. In fact, participant 134 (Figure S1b) does not have associations between physical activity and affect at all. Participants 228 (Figure S1c) and 296 (FigureS1d) have associations between ENMO and NA, although varying in timing and sign. That is, participant 228 has a negative lagged association between ENMO and NA, indicating that higher ENMO was associated with *decreased* subsequent NA 3 hours later. In contrast, participant 296 has a positive contemporaneous association between ENMO and NA, indicating that higher ENMO was associated with higher NA within the 3-hour time window.

| 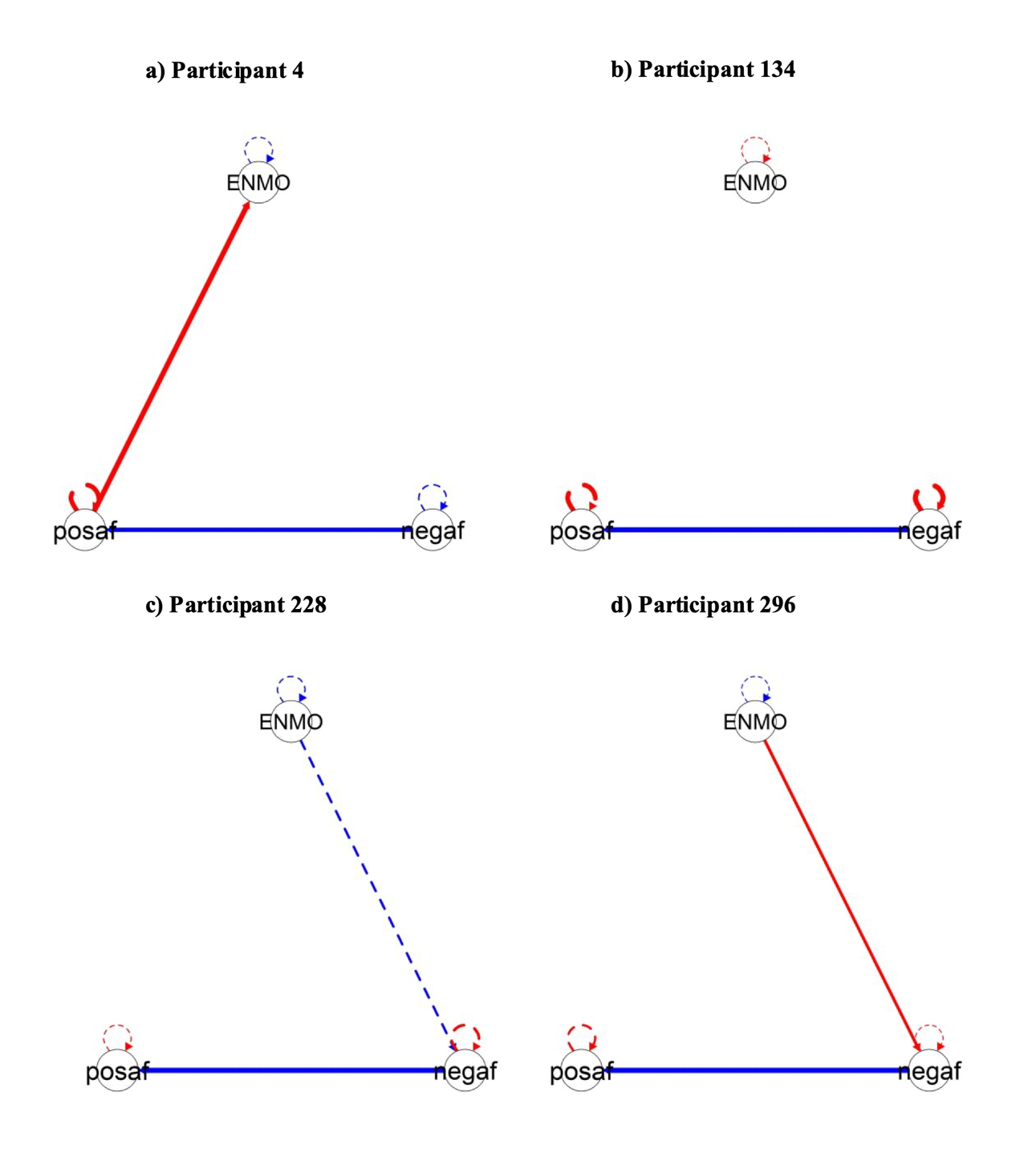 |
| --- |
| **Figure S1**: Sample individual person-specific models generated by confirmatory CS-GIMME. posaf = positive affect, negaf = negative affect. Red paths are positive, blue paths are negative, dashed lines are lagged relationships of lag-1 (autoregressive effects depicted above each variable node), and solid lines are contemporaneous from the same timepoint. Line thickness corresponds to strength of the effect. |

### S-GIMME for Data-driven Subgroups

We conducted the completely data-driven S-GIMME to identify putative data-driven subgroups that share associations between variables A total of 297 (out of 300) person-specific models fit the data well according to the fit mean indices (RMSEA = .02, NNFI = .60, CFI = .99, SRMR = .06).^[[1]](#footnote-1)^ The models of 3 individuals did not meet the criteria for excellent fit (RMSEA = .09, NNFI = .91, CFI = .96, SRMR = .08). These 3 individuals were excluded for further analyses. All detected associations are described in Table S1.

***Group-level results.*** In contrast to the confirmatory CS-GIMME findings, S-GIMME did not identify any group-level associations, indicating that there was no association between variables that was recovered in more than 75% of the full sample.

***Subgroup-level results.*** The data-driven subgrouping resulted in four subgroups (*n*_1_ = 39, *n*_2_ = 93, *n*_3_ = 65, *n*_4_ = 5) and 95 singletons. These subgroup-specific associations showed associations between PA and NA, but not between physical activity and PA or NA. In total, 95 individuals could be not be categorized into these four subgroups and are considered as outliers (Lane et al., 2019). The modularity value of the subgroup solution was low (Q = 0.079).

The subgroups were evaluated by conducting three tests (see Supplementary Methods section) that compared the modularity value to a distribution of random solutions after perturbing the weights of the paths (Gates et al., 2019). The modularity value of the subgroup solution was low but robust according to the modularity test (*Qsolution* = 0.079 > *Q*95 = 0.049). The subgroups were moderately robust according to the Adjusted Rand Index (ARI = 0.799, α > 0.20, significantly higher than the random reassignment benchmark of ARI = 0.737, *t*(99) = 6.37, *p* < .001), but not the Variation of Information (VI = 1.360, α > 0.20). The VI indicates that 19% of the subgroup assignments need to be changed in order to be as different as when 20% of participant subgroup assignments are randomly switched. These results indicate no stable data-driven subgroup solution. As these tests were no indication of a robust subgroup solution, we did not compare the data-driven subgroups on characteristics (see Method section).

***Individual-level results.*** S-GIMME recovered the same individual-level associations between activity and affect as confirmatory CS-GIMME. An overview of the recovered paths in S-GIMME can be found in Table S1. As in the associations derived from confirmatory CS-GIMME, the associations vary widely in strength (i.e., beta coefficients), sign (i.e., positive or negative), timing (i.e., contemporaneous and lagged) and direction. With regard to activity-affect dynamics, the models of 136 of 297 individuals (45.8%) contained at least one significant association (*p <* .05) between ENMO and affect (either PA or NA), with the most common association being the positive contemporaneous association between ENMO and PA (25.5%). As in CS-GIMME, S-GIMME detected more individuals with contemporaneous associations between physical activity and affect (33%) than lagged associations (17.2%) and more individuals with person-specific associations between physical activity and PA (34%) than NA (16.5%).

|  | | **Table S1:** The total number of individuals and mean beta coefficients per detected association between ENMO, positive affect, negative affect for data-driven S-GIMME (*n* = 297) | | | | | | | | | |  |
| --- | --- | --- | --- | --- | --- | --- | --- | --- | --- | --- | --- | --- |
|  | |  |  |  | Frequency | | | Beta coefficient | | | | |
|  | | Association | | | Total *n* | *n* positive beta | *n* negative beta | Mean | *SD* | Min | Max | |
| **Subgroup** | | | | |  |  |  |  |  |  |  | |
| 1 | | - |  | - | 39 | - | - | - | - | - | - | |
| 2 | | NA*_t_* | - | PA*_t_* | 93 | 0 | 93 | -0.703 | 0.097 | -0.896 | -0.327 | |
| 3 | | PA*_t_* | - | NA*_t_* | 65 | 2 | 63 | -0.683 | 0.190 | -0.906 | 0.156 | |
| 4 | | PA*_t_* | - | NA*_t_* | 5 | 0 | 5 | -0.323 | 0.143 | -0.440 | -0.077 | |
| **Person-specific** | | | | |  |  |  |  |  |  |  | |
|  | | ENMO*_t_* | – | PAt*_t_* | 76 | 71 | 5 | 0.295 | 0.211 | -0.530 | 0.592 | |
|  | | ENMO*_t_* | – | NA*_t_* | 25 | 19 | 6 | 0.206 | 0.362 | -0.409 | 1.144 | |
|  | | PA*_t-1_* | 🡪 | ENMO*_t_* | 20 | 14 | 6 | 0.183 | 0.386 | -0.423 | 1.010 | |
|  | | PA*_t-1_* | 🡪 | NA*_t_* | 18 | 8 | 10 | -0.151 | 0.531 | -0.935 | 0.487 | |
|  | | NA*_t-1_* | 🡪 | PA*_t_* | 17 | 7 | 10 | -0.124 | 0.425 | -0.719 | 0.466 | |
|  | | ENMO*_t-1_* | 🡪 | NA*_t_* | 14 | 5 | 9 | -0.030 | 0.390 | -0.412 | 0.895 | |
|  | | NA*_t-1_* | 🡪 | ENMO*_t_* | 12 | 6 | 6 | -0.002 | 0.419 | -0.470 | 0.515 | |
|  | | ENMO*_t-1_* | 🡪 | PAt*_t_* | 11 | 7 | 4 | 0.069 | 0.329 | -0.447 | 0.431 | |
| *Note.* NA = Negative Affect, PA = Positive Affect. *SD* = standard deviation of the beta.  Total *n* indicates the total number of individuals for whom each association is included in the model. *n* positive beta and *n* negative beta indicate the number of individuals with positive and negative associations, respectively. Underscript t-1 denotes a lagged association. Contemporaneous associations identified by GIMME are presented without directional interpretation and aggregated accordingly. The person-specific association between PA and NA was omitted due to the significant subgroup-level associations. Rows are ordered by frequency of detected associations. | | | | | | | | | | |  |  |

**References**

Gates, K. M., Fisher, Z. F., Arizmendi, C., Henry, T. R., Duffy, K. A., & Mucha, P. J. (2019). Assessing the robustness of cluster solutions obtained from sparse count matrices. *Psychological Methods*, *24*(6), 675–689. doi: 10.1037/met0000204

Karrer, B., Levina, E., & Newman, M. E. J. (2008). Robustness of community structure in networks. *Physical Review E*, *77*(4), 046119. doi: 10.1103/PhysRevE.77.046119

Lane, S. T., & Gates, K. M. (2017). Automated Selection of Robust Individual-Level Structural Equation Models for Time Series Data. *Structural Equation Modeling: A Multidisciplinary Journal*, *24*(5), 768–782. doi: 10.1080/10705511.2017.1309978

Lane, S. T., Gates, K. M., Pike, H. K., Beltz, A. M., & Wright, A. G. C. (2019). Uncovering general, shared, and unique temporal patterns in ambulatory assessment data. *Psychological Methods*, *24*(1), 54–69. doi: 10.1037/met0000192

Pons, P., & Latapy, M. (2005). Computing Communities in Large Networks Using Random Walks. In pInar Yolum, T. Güngör, F. Gürgen, & C. Özturan (Eds.), *Computer and Information Sciences—ISCIS 2005* (pp. 284–293). Berlin, Heidelberg: Springer. doi: 10.1007/11569596_31

Zwieten, N. van, Aalbers, G., Riese, H., Lamers, F., Hillegers, M. H. J., & Penninx, B. (2023). *Group, subgroup and person-specific longitudinal associations between affect and physical activity in individuals with depressive and/or anxiety disorders and healthy controls: An ambulatory assessment study*. Retrieved from https://osf.io/yu4cf

1. The NNFI fit index of one individual was extremely low (-138), causing the mean NNFI fit index to be lower than what is qualified as excellent fit (NNFI > .95). However, the other fit indices for this individual were excellent, hence we did not exclude this individual from further analyses. [↑](#footnote-ref-1)
